# Kaempferol inhibits oxidative stress and reduces macrophage pyroptosis by activating the NRF2 signaling pathway

**DOI:** 10.1371/journal.pone.0325189

**Published:** 2025-06-06

**Authors:** Yu Wang, Chaofan Chen, Yushan Li, Ran Li, Jinghan Wang, Chao Wu, Haonan Chen, Yingchao Shi, Shengfang Wang, Chuanyu Gao

**Affiliations:** 1 School of Physical Education, Henan University, Kaifeng, China, Kaifeng, China; 2 Henan Key Lab for Prevention and Control of Coronary Heart Disease, Zhengzhou, China; 3 Fuwai Central China Cardiovascular Hospital, Zhengzhou, China; Sungkyunkwan University - Suwon Campus: Sungkyunkwan University - Natural Sciences Campus, KOREA, REPUBLIC OF

## Abstract

Kaempferol exhibits various biological activities, including antioxidant and anti-inflammatory effects. Its role in modulating lipid metabolism and inhibiting inflammatory responses to suppress the progression of atherosclerosis has been confirmed. However, its impact on macrophage pyroptosis and the underlying mechanisms remain unclear. This study aims to investigate the effects of kaempferol (Kae) on lipopolysaccharide (LPS)-induced macrophage pyroptosis and its potential mechanisms. In the experiments, we used the CCK8 assay to evaluate cell viability, ROS detection kits to measure intracellular reactive oxygen species (ROS) levels, Western Blot to detect the expression of proteins such as NOD-like receptor family pyrin domain-containing 3 (NLRP3), nuclear factor erythroid 2-related factor 2 (NRF2), gasdermin D (GSDMD), and heme oxygenase-1 (HO-1), and immunofluorescence to observe NRF2 nuclear translocation. The results showed that kaempferol alleviated LPS-induced cell viability decline and lactate dehydrogenase (LDH) release, inhibited excessive ROS generation, and suppressed NLRP3 inflammasome activation by increasing glutathione (GSH) and HO-1 levels, thereby reducing the expression of inflammatory factors. Additionally, kaempferol promoted NRF2 nuclear translocation, and the application of the NRF2 inhibitor ML385 reversed its antioxidant and anti-inflammatory effects. In vivo experiments further confirmed that kaempferol inhibited oxidative stress and reduced macrophage pyroptosis by activating the NRF2 pathway.

## Introduction

Atherosclerosis (AS) is a chronic inflammatory cardiovascular disease [1]. It causes approximately 4 million deaths annually worldwide, with related deaths expected to exceed 23.6 million by 2030 [2]. Inflammation is a core mechanism in the development of AS [3]. Studies have shown that macrophages constitute a major part of plaques [4]. In the early stages, they inhibit plaque formation by clearing lipoproteins and apoptotic cells, but excessive cell death in later stages promotes inflammation and the formation of large necrotic lipid cores, significantly increasing the risk of plaque rupture. Although current pharmacological treatments and surgical interventions have shown some efficacy in managing atherosclerosis, they have significant limitations [5]. Long-term medication may cause side effects such as liver damage and bleeding, while surgical interventions carry the risk of restenosis [6,7]. Therefore, there is an urgent need to explore new treatments for atherosclerosis. Innovations that improve treatment outcomes, enhance patient compliance, and improve prognosis would be particularly valuable [8,9].

Macrophage pyroptosis increases the risk of AS plaque rupture [10]. Pyroptosis is closely related to the occurrence and progression of AS, serving as a significant cause of inflammatory responses in the body and playing a key role in the pathological process of atherosclerosis [11]. Macrophage pyroptosis is an inflammatory cell death mode closely associated with the instability of atherosclerotic plaques. Research has shown that nicotine induces macrophage pyroptosis by increasing ROS, characterized by NLRP3 inflammasome assembly, caspase-1 cleavage, and increased production of IL-1β, IL-18, and GSDMD [12]. Wang et al [13] demonstrated that PBBQ metabolites exacerbated AS by activating NLRP3 inflammatory vesicles through CD36-mediated lipid accumulation. Inhibiting NLRP3 expression and reducing macrophage pyroptosis can stabilize AS plaques [14]. Targeting macrophage pyroptosis is an effective strategy for stabilizing AS, and developing small molecule inhibitors to block pyroptosis is a potential research direction.

Kaempferol is a polyphenolic compound with potential anti-atherosclerotic properties [15]. In recent years, it has garnered increasing attention due to its potential anti-inflammatory properties. Preclinical studies have shown that kaempferol exhibits a wide range of biological activities, including antioxidant, anti-inflammatory, antibacterial, anticancer, cardiovascular protection, neuroprotection, and antidiabetic effects [16]. In vitro studies have shown that kaempferol induces antioxidant effects and increases antioxidant enzyme expression by scavenging free radicals and inhibiting reactive oxygenase activity [17]. It also potentially prevents diseases associated with oxidative stress by scavenging superoxide anion [18]. Kaempferol has significant anti-inflammatory effects, regulating the activity of pro-inflammatory enzymes and the expression of inflammatory genes, inhibiting the production of inflammatory factors, and alleviating inflammation [19]. Kaempferol has been shown to significantly inhibit the MAPK pathway in LPS-induced human monocytes (THP-1), leading to reduced production of inflammatory factors such as macrophage-derived chemokine (MDC) and interleukin-8 (IL-8), effectively alleviating inflammation [20]. Additionally, kaempferol can mitigate vascular inflammatory responses, indicating its potential in preventing atherosclerosis. However, whether kaempferol can improve mechanisms related to macrophage activation remains unclear.

Oxidative stress is widely recognized as a significant activator of NLRP3 [21]. Studies have shown that that NLRP3 triggers inflammatory vesicle activation and initiates programmed death process by sensing abnormal accumulation of ROS [21–23]. NRF2, as a core transcription factor, maintains redox homeostasis by regulating the expression of antioxidant enzyme genes such as HO-1 and superoxide dismutase (SOD) [24]. Previous research has shown that exogenous activation of NRF2 can reduce intracellular ROS levels, thereby inhibiting pyroptosis [25]. Activation of the NRF2 pathway is beneficial to cells in the early stages of oxidative stress, as it stimulates the production of antioxidant enzymes that help reduce intracellular ROS levels [26,27]. Furthermore, NRF2 is crucial for regulating cellular homeostasis and preventing various forms of cell death, including ferroptosis and pyroptosis [28].

This study evaluated the effects of kaempferol on LPS-induced THP-1 macrophage proliferation and its impact on macrophage pyroptosis. The results showed that kaempferol significantly inhibited excessive ROS generation by promoting NRF2 nuclear translocation, increased GSH content, and enhanced HO-1 expression, thereby suppressing NLRP3 inflammasome activation. Additionally, we further explored its potential regulatory mechanisms in pyroptosis, with a particular focus on the involvement of NRF2.

## 2. Materials and methods

### 2.1. Reagents

Kaempferol is provided by Shanghai Aladdin Biochemical Technology Co., Ltd., with purity ≥ 98% (product number: 492-27-3). Lipopolysaccharides are provided by Shanghai Haoyuan Chemexpress Co., Ltd. (HY-D1056). Phorbol esters (PMA) are provided by Beijing Solarbio Science & Technology Co., Ltd. (P6741). The ML385 inhibitor is purchased from Shanghai Haoyuan Chemexpress Co., Ltd. (846557-71-9).

### 2.2. Experimental cells

THP-1 monocytes were used (Wuhan Pricella Biotechnology Co., Ltd.). The cells were cultured with 1640 medium containing 10% fetal bovine serum at 37°C in an incubator with 5% CO₂ The culture medium was changed every 1 day. Before the experiments, THP-1 monocytes were treated with fobol ester 100nM for 48h to differentiate them into macrophages for subsequent experiments [29].

### 2.3. Cell culture

#### 2.3.1. Cell resuscitation.

Before removing the cells from the liquid nitrogen tank, wipe the desktop of the ultra-clean table and the pipette gun rod with alcohol and sterilize the ultra-clean table with ultraviolet light for 30 min. After sterilization, remove the cell cryopreservation tubes from the liquid nitrogen tank, place them in the 37°C thermostatic water bath to melt rapidly in 1 min, wipe the outside of the cryopreservation tubes with an alcohol swab to disinfect the outside of the tubes, then transfer them to the sterile ultra-clean table. Transfer the cell cryopreservation solution to a 15 ml centrifuge tube and centrifuge at 1000 rpm for 5 min, discard the supernatant and add 2 ml of fresh complete medium to resuspend the cells, and then transfer the cell suspension into T25 cell culture flasks, with the total amount of medium in each flask being 5 ml. Place the flasks into an incubator at 37°C and 5% CO₂ for incubation. On the next day, observe the cell status and replace the fresh medium. Allow them to grow to their full size and perform passaging.

#### 2.3.2. Cell passage.

Cells were passaged when they reached 80%−90% cell growth. After sterilizing with alcohol, T25 cell culture flasks were placed in an ultra-clean table, and the cells were passaged in a half-liquid exchange manner. The cell culture flasks were shaken slightly to homogenize the cells, and the cells were evenly divided into two T25 culture flasks in a ratio of 1:2, and then the flasks were erected. Each flask was replenished with an equal amount of medium up to 5 ml, and placed in an incubator at 37°C and 5% CO₂ for incubation. The following day, the cell growth status was observed. On the following day, the cell growth status was observed.

#### 2.3.3. Cell cryopreservation.

Collect the cells with good growth status in 15 ml centrifuge tube, with a typical cell concentration of 5 × 10^6^-5 × 10^7^/ml. Transfer the cell suspension into the centrifuge tube and put it into centrifuge at 1000 rpm for 5 min, after that, discard the supernatant, add 1 ml of cryopreservation solution (containing 10% DMSO, CST,#12611). Gently resuspend the cells by pipetting, then transfer the cell suspension into 1 ml cryopreservation tubes, Place the tubes in a gradient cooling box, transfer it into a – 80°C refrigerator for 2–3 days before re-transferred to liquid nitrogen tank for long-term storage.

#### 2.3.4. Cell Count.

Using the blood cell counting method to count cells, the number of cells used in each experiment is kept consistent, that is, in the Western Blot experiment, the number of cells in each group reaches 1x10^6^/ml, and in the CCK-8 experiment, the number of cells reaches 1x10^5^/ml.

#### 2.3.5. Cell damage model and grouping.

Mainly using LPS to treat macrophages, LPS composed primarily of lipids and polysaccharides, is a substance that can activate immune cells attached to the surface of Gram-negative bacteria [24]. It exerts its effects mainly by binding to TLR4 receptors on the surface of phagocytes, triggering the activity of phagocytes (including NK cells and T cells) to enhance the body’s immune system. When LPS interacts with macrophages, it activates intracellular signaling pathways lead to inflammations, oxidative stress, and ultimately leading to cell damage and pyroptosis. Macrophages are mainly divided into 5 groups: i) Control group (DMSO) [30], ii) LPS group (1 μg/ml LPS for 4 hours) [31], iii) Kae + LPS group (100µM Kae for 2hours, then 1 μg/ml LPS for 4 hours). iv): ML385 + Kae + LPS (5µM ML385 treated for 1hour, 100µM Kae treated for 2hours, and then 1 μg/ml LPS treated for 4 hours), v): ML385 inhibitors were treated as a separate group [32].

### 2.4. Experimental methods

#### 2.4.1. Cell proliferation analysis.

Macrophages were cultured in 96-well plates (100,000 cells per well), and the cell counting kit-8 working solution (CCK-8) (Beyotime Institute of Biotechnology, C0037) was configured by diluting the medium at a concentration of 1:10.A total of 100 microliters of the working solution was added to each well, and continuing to incubate for 1h in a cell culture incubator at 37°C, Absorbance was measured at 450nm with an enzyme meter in order to detect the proliferative capacity of the cells for cell proliferation ability detection.

#### 2.4.2. Hoechst/PI staining.

Macrophages were inoculated into a 48 well plate at a density of 20000 cells per well. After treating the macrophages, the culture medium was aspirated, and the cells were rinsed with PBS three times at room temperature, each time for 3 minutes. Next, 5 microliters of Hoechst staining solution (Beyotime Institute of Biotechnology, COO3) were added to the aspirated liquid and stained for 5 minutes. Afterward, 5 microliters of PI staining solution were added and the cells were incubated at 4° C for 25 minutes. After incubation, the staining solution was removed, and the cells were rinsed with PBS three times, each for 3 minutes. After removing the liquid, a drop of anti-fluorescence quenching sealing solution was dropped on a glass slide, and the cell crawler with cells attached to it was covered, ensuring the cells to contacted with the mounting medium without trapping air bubbles. The macrophages were observed under a 200x fluorescence microscope and images were recorded. To calculating the percentage of PI positive cells, the number of PI positive cells/total number of Hoechst positive cells was used. Three randomly selected regions were used for this calculation.

#### 2.4.3. Determination of malondialdehyde (MDA), glutathione (GSH), and superoxide dismutase (SOD).

Inoculate macrophages were seeded into a 6-well plate at a density of 1000000 cells per well. Extract macrophage lysate was provided according to manufacturer’s instructions the manufacturer and measure MDA, GSH, and SOD levels (the kit comes from Beyotime Institute of Biotechnology, S0131S, S0053, S0101S). Subsequently, the absorbance values were analyzed using a microporous spectrophotometer (MDA measured absorbance at 532nm, GSH measured absorbance at 412nm, SOD measured absorbance at 450nm).

#### 2.4.4. Determination of lactate dehydrogenase (LDH).

Macrophages were inoculated into 96-well plates with 10,000 cells per well. After cell processing, the cell culture plates were centrifuged at 400g in a multiwell plate centrifuge at 4°C for 5 min, the supernatant was aspirated, and 150μl of the LDH-releasing reagent provided in the kit Beyotime Institute of Biotechnology, C0016) diluted 10-fold in PBS was added, and the cells were shaken well and placed into an incubator for continued incubation for 1h, remove and put into a multiwell plate centrifuge, 400g, 4°C centrifugation for 5 min, after that, take 120 μL of supernatant from each well respectively, add to a new 96-well plate, and measure the absorbance at 490nm.

#### 2.4.5. Western Blot for protein immunoblotting.

The cell samples were fully lysed by RIPA buffer lysis (Beyotime Institute of Biotechnology, abs9230−100 ml). The lysate was collected after 30 min, and placed in a centrifuge at 4°C, 14,000g, for 15 min for centrifugation. After centrifugation, the supernatant was used in the BCA Protein Quantification Kit (BCA) (absin, abs9232) method to determine the protein concentration. Then SDS-PAGE protein uploading buffer (5X) (Beyotime Institute of Biotechnology, P0015L) was added to the samples, which were then heated in a water bath at 100°C for 10 min, with 40 μg of protein on each lane were separated on a 10% SDS-PAGE gel. Electrophoresis was performed at constant voltages (80V, 30 min upper gel, 120V, 80 min lower gel), and transferring the membrane utilizing a semi-dry transfer device (Bio-Rad Laboratories, constant pressure, 15V, 30 min.) to separate the same amounts of proteins on a 0.22μm polyvinylidene fluoride (PVDF) membrane. At the end of membrane transfer, the membrane was washed 3 times with TBST for 5 min. Subsequently, the membrane was closed, and the membrane was closed for 2h. At the end of the closure, the membrane was washed 3 times with TBST for 5 min each, the primary antibody was incubated overnight in a 4°C refrigerator, and at the end of the primary antibody incubation, the membrane was washed 3 times with TBST for 5 min, followed by the secondary antibody incubation, and at the end of the incubation, the membrane was washed 3 times again. Color development was performed with enhanced chemiluminescence development reagent, and the intensity of the bands was quantified using ImageJ software to normalize the expression level of the target proteins to the intensity of the bands of the total proteins. Antibodies included NLRP3(1:1000, abcam, ab263899), GSDMD(1:1000, abcam, ab215203), Caspase-1(1:1000, abcam, ab207802), Tubulin(1:1000, MyBiosource, MBS854064), Histtone H3(1:1000, abcam, abs171823), NRF2(1:1000), HO-1(1:1000, abcam, ab52947), and the secondary antibody was goat anti-rabbit IgG (H + L) (1:10,000, Beyotime Institute of Biotechnology, A0208).

#### 2.4.6. Immunofluorescence.

Inoculate macrophages into a 24 well plate at a density of 50,000 cells per well. After processing, the cells were allowed to form a monolayer before carefully removing the cell slides to prevent detachment. The slides were then washed 3 times with sterile PBS at room temperature for 5 min each. Permeabilization was performed using 0.3% Triton X-100 in PBS (or TBST) for 5 minutes at room temperature, followed by three additional PBS washes, each for 5 minutes. After blotting the PBS on absorbent paper, the slides were blocked with 5% bovine serum albumin for 60 min. From this point onward, all steps were performed in a humidified environment to prevent drying of the samples. After blocking, the slides were washed again and incubated with diluted primary antibody (anti-NRF2, 1:200, Cell Signaling Technology, 12721) in a humid chamber at 4°C for 12 hours. Following primary antibody incubation, the antibody solution was recovered, and the slides were washed three times with PBST on a shaker for 5 minutes each. Next, the slides were incubated with a diluted fluorescent secondary antibody (1:500, abcam, 150077) was added in a wet box for 2h. After secondary antibody incubation, the slides were washed three times with PBST for 5 minutes each. Since fluorescence is sensitive to light, all subsequent steps, starting with the addition of the fluorescent secondary antibody, were performed under light-protected conditions. DAPI (Beyotime Institute of Biotechnology, C1005) was added dropwise to slides at room temperature, and the cells were re-stained for 10 min, protected from light. The cells were re-stained for 10 min at room temperature, and the cells were washed with PBST for 3 times, each time for 5 min, to wash away the excess DAPI. The slides were gently blotted dry with absorbent paper and mounted with an anti-fade mounting solution. The samples were observed using a laser confocal microscope (LSM 800), and images were captured at 640x magnification for preservation.

#### 2.4.7. Cell nucleus separation.

Macrophages were inoculated into a 6-well plate at a density of 1000000 cells per well. After cell processing, the cell nuclei were separated using the nuclear protein and cytoplasmic protein extraction kit (Beyotime Institute of Biotechnology, P0028) ollowing the manufacturer’s instructions. The supernatant containing nuclear protein was collected.

#### 2.4.8. Intracellular ROS detection.

Macrophages were inoculated into 96-well plates with 10,000 cells per well, and after the cells were processed, DCFH-DA was diluted 1,000-fold using PBS according to the instructions of the DCFH-DA kit (Beyotime Institute of Biotechnology, S0033S). A total of 100 µl of the working solution was added to each well, and the cells were incubated at 37°C for 20 minutes. After incubation, the cells were washed three times with PBS, with each wash lasting 5 minutes. At the end, ROS intensity was observed under a fluorescence microscope at x200 magnification, using an excitation wavelength of 488nm and emission wavelength of 525nm.

#### 2.4.9 Statistical analysis.

All experimental data in this paper are expressed as mean and standard deviation, and each experiment was repeated at least three times, plotted using Graph Pad Prism 9.0 software, and statistically analyzed by t-test. Statistical significance was achieved when the condition of significant difference was *P* < 0.05.

## 3. Research results

### 3.1. Kaempferol can enhance macrophage activity

**Fig1:**
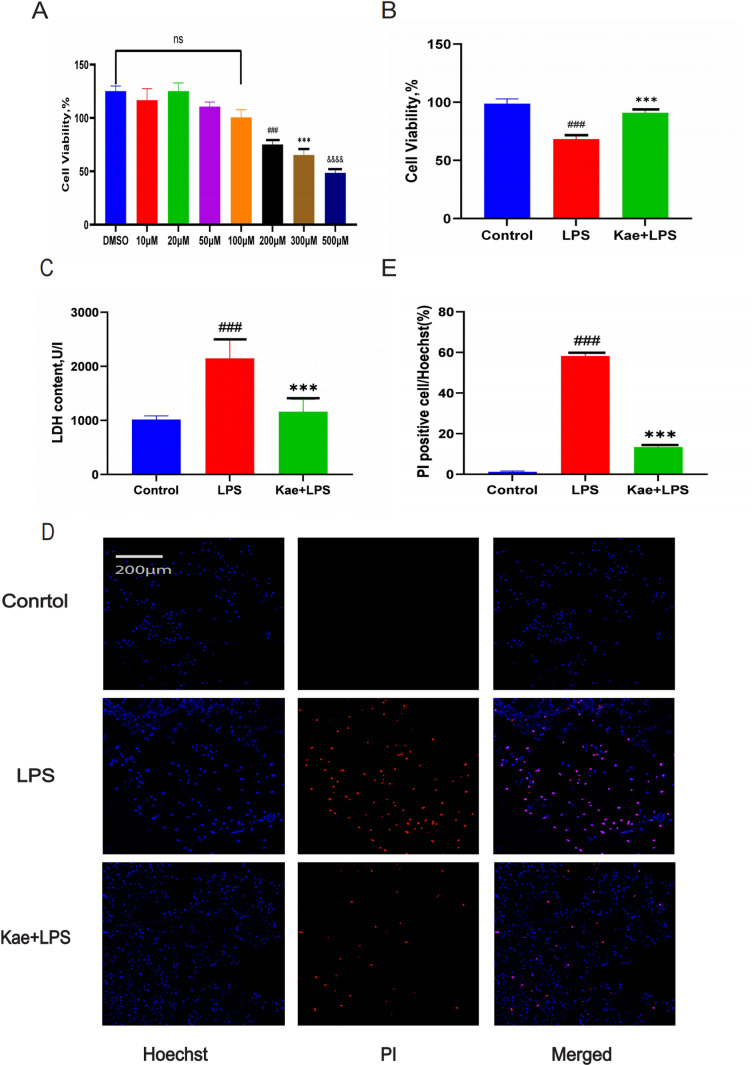
Kaempferol can enhance LPS induced macrophage activity. Cells are divided into three groups. Control group; LPS group; Kae + LPS group, using 1 μg/mL LPS for 4 h; Kae + LPS group, using 100 µM Kae treated macrophages for 2h, followed by 1 μg/ml LPS for 4 h. (A) CCK-8 detection of kaempferol concentration results. (“###” The symbol indicates the comparison between the 200µM Kae and the DMSO group. “***” The symbol indicates the comparison between the 300µM Kae and the DMSO group. “&&&&” The symbol indicates the comparison between the 500µM Kae and the DMSO group.)(B) CCK-8 assay results for the viability of three groups of macrophages.(C) Results from the lactate dehydrogenase (LDH) assay kit.(D) Results from Hochst/PI double staining.”###”The symbol indicates the comparison between the LPS group and the Control group, with a P-value of <0.001.”***”The symbol indicates the comparison between the Kae + LPS group and the LPS group, with a P-value of <0.001.The significance levels are denoted as follows:”#” represents P < 0.05,”##” represents P < 0.01”###” represents P < 0.001,”*” represents P < 0.05,”**” represents P < 0.01,”***” represents P < 0.001,”&” represents P < 0.05,”&&” represents P < 0.01,”&&&” represents P < 0.001,”&&&&” represents P < 0.0001.The experimental results were independently repeated three times to ensure the reliability and consistency of the data. Each experiment was conducted under the same conditions and methods to minimize random errors. Kae: Kaempferol; LPS: Lipopolysaccharides; LDH: lactate dehydrogenase; Hochst staining; PI: Propidium iodide. The error bars shown in the figure represent a 95% confidence interval.

In this study, macrophages were treated with a concentration of 1 µg/mL LPS for 4h [31]. To determine the optimal concentration of kaempferol to be used in the subsequent experiments, macrophages were treated with different concentrations of kaempferol at 0, 20, 50, 100, 200,300,500µM, and use the carrier (0.1% DMSO treatment) separately as a control. no significant inhibitory effect on macrophage activity was detected below 100µM kaempferol using the cell proliferation assay CCK-8 (Fig 1A). As a result, 100 µM of kaempferol was selected for a 2-hour intervention in the next phase of the experiment [30,33]. Cell proliferation capacity was assayed using CCK-8 and 1 µg/mL LPS induced a significant decrease in macrophage viability compared to the control group. Meanwhile, kaempferol pretreatment was found to increase macrophage activity compared with the macrophage group intervened with LPS alone (Fig 1B). Intracellular release of LDH is a key feature, and by using lactate dehydrogenase (LDH) assay of cells it was found that kaempferol pretreatment reduced LDH levels in the supernatant of the LPS group (Fig 1C). And the Hochst/PI double staining method was used to analyze the protective effect of kaempferol on LPS induced macrophages (Fig 1D). The results showed that pre-treatment with kaempferol could reduce the level of PI positive cells in LPS induced macrophages (Fig 1E). All these data indicate that kaempferol can improve macrophage survival rate.

### 3.2. Kaempferol can inhibit LPS-induced inflammatory response and NLRP3 inflammasome activation, and improve pyroptosis

**Fig 2:**
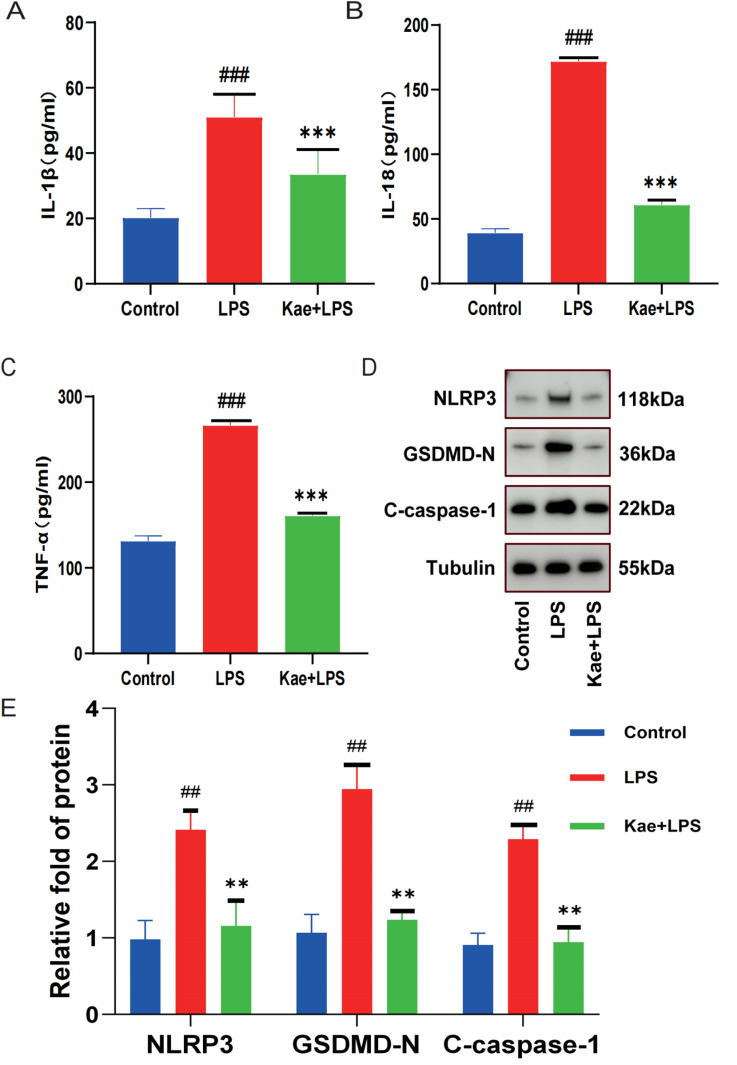
Kaempferol can alleviate the inflammatory response induced by LPS in macrophages and improve cell pyroptosis. Cells are divided into three groups. Control group; LPS group; Kae + LPS group, using 1 μg/mL LPS for 4 h; Kae + LPS group, using 100 µM Kae treated macrophages for 2h, followed by 1 μg/ml LPS for 4 h. Detect the expression level of inflammatory cytokines in the cell supernatant. (A) Detection of the expression level of the inflammatory cytokine IL-1β in cell supernatants using an ELISA kit.(B) Detection of the expression level of the inflammatory cytokine IL-18 in cell supernatants using an ELISA kit.(C) Detection of the expression level of the inflammatory cytokine TNF-α in cell supernatants using an ELISA kit.(D) Detection of the expression levels of NLRP3, GSDMD, and Caspase-1 proteins in macrophages using Western Blot.(E) Quantification of NLRP3, GSDMD, and Caspase-1 proteins using Image J.“###”The symbol indicates the comparison between the LPS group and the Control group, with a P-value of <0.001.”***”The symbol indicates the comparison between the Kae + LPS group and the LPS group, with a P-value of <0.001.The significance levels are denoted as follows:“#” represents P < 0.05,”##” represents P < 0.01”###” represents P < 0.001,”*” represents P < 0.05,”**” represents P < 0.01,”***” represents P < 0.001,”&” represents P < 0.05,”&&” represents P < 0.01,”&&&” represents P < 0.001.The experimental results were independently repeated three times to ensure the reliability and consistency of the data. Each experiment was conducted under the same conditions and methods to minimize random errors. Kae: Kaempferol; LPS: Lipopolysaccharides.NLRP3: NOD-like receptor protein 3; GSDMD: Gasdermin-D. The error bars shown in the figure represent a 95% confidence interval.

To assess that kaempferol could inhibit LPS-induced inflammatory response and the activation of NLRP3 inflammatory vesicles, the expression levels of inflammatory factors such as IL-1β, IL-18, and TNF-a were detected according to ELISA kits. The results demonstrated that kaempferol intervention significantly reduced the levels of IL-1β, IL-18, and TNF-α in the cell supernatant (Fig 2A–C). Additionally, the protein expression levels of NLRP3, Caspase1, and GSDMD were detected by protein immunoblotting WB. Compared with the Control group, it was found that the expression levels of the above three types of proteins in the LPS group were significantly increased, while the expression levels of the three types of proteins were significantly decreased after the intervention of kaempferol (Fig 2D–E). The above data showed that kaempferol could significantly inhibit the activation of NLRP3 inflammatory vesicles and improve macrophage pyroptosis.

### 3.3. Kaempferol can reduce LPS induced oxidative stress in macrophages

**Fig 3:**
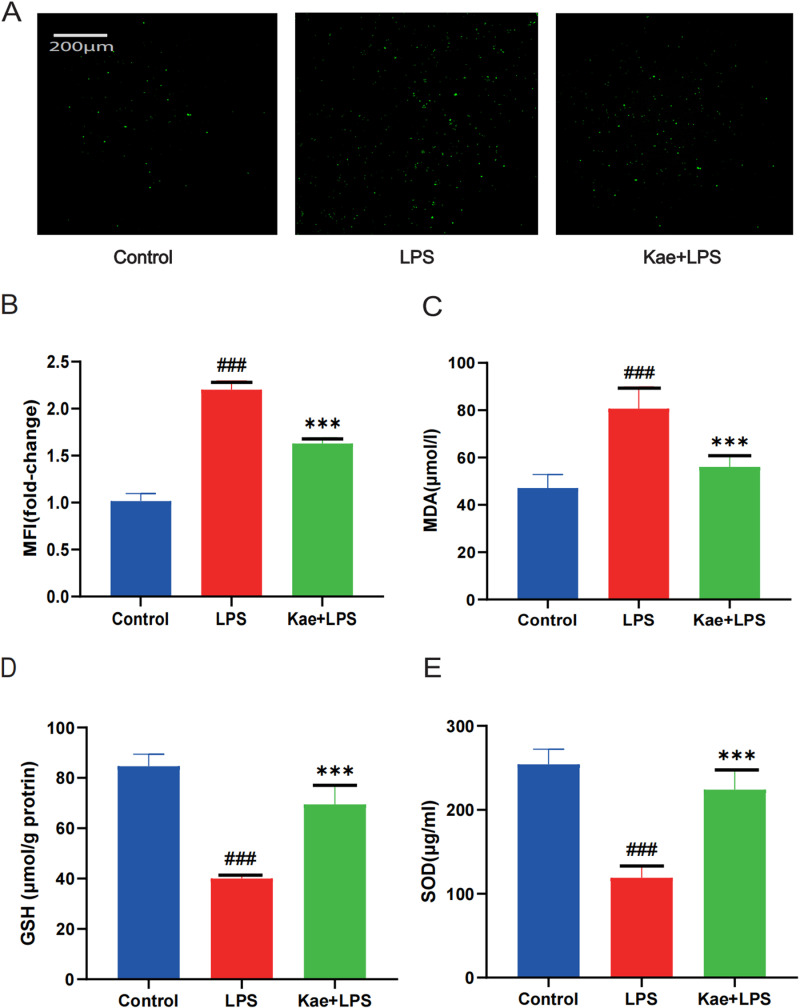
Kaempferol can alleviate LPS induced oxidative stress in macrophages. Cells are divided into three groups. Control group; LPS group; Kae + LPS group, using 1 μg/mL LPS for 4 h; Kae + LPS group, using 100 µM Kae treated macrophages for 2h, followed by 1 μg/ml LPS for 4 h. (A) Analyze the total ROS levels in cells using the DCFH-DA probe,(B) Perform quantitative analysis of cellular ROS expression using Image J,(C) Detect the expression levels in different groups using the MDA assay kit,(D) Measure the expression levels in different groups using the GSH assay kit,(E) Assess the expression levels in different groups using the SOD assay kit.“###”The symbol indicates the comparison between the LPS group and the Control group, with a P-value of <0.001.”***”The symbol indicates the comparison between the Kae + LPS group and the LPS group, with a P-value of <0.001.The significance levels are denoted as follows:“#” represents P < 0.05,”##” represents P < 0.01”###” represents P < 0.001,”*” represents P < 0.05,”**” represents P < 0.01,”***” represents P < 0.001,”&” represents P < 0.05,”&&” represents P < 0.01,”&&&” represents P < 0.001.The experimental results were independently repeated three times to ensure the reliability and consistency of the data. Each experiment was conducted under the same conditions and methods to minimize random errors. Kae: Kaempferol; LPS: Lipopolysaccharides; SOD: Superoxide dismutase; MDA: Malondialdehyde; GSH: Glutathione; ROS: Reactive oxygen species. The error bars shown in the figure represent a 95% confidence interval.

Oxidative stress is a potent activator of NLRP3 inflammatory vesicles. To confirm whether kaempferol could reduce the level of oxidative stress, DCFH-DA staining was used to analyze the level of ROS in macrophages, and it was found that kaempferol significantly reduced the level of ROS in macrophages compared to the LPS-alone group (Fig 3A–B).

Malondialdehyde (MDA) is a metabolite of lipid peroxidation in cell membranes and MDA levels represent intracellular lipid peroxidation levels. Examination of both revealed that kaempferol decreased MDA levels in the LPS group (Fig 3C). In addition, kaempferol increased GSH levels and SOD activity (Fig 3D–E). These data suggest that kaempferol improved the level of oxidative stress after LPS induction by inhibiting ROS production and increasing the activity of antioxidant proteins.

### 3.4. Kaempferol can enhance the activity of NRF2 in macrophages.

**Fig 4:**
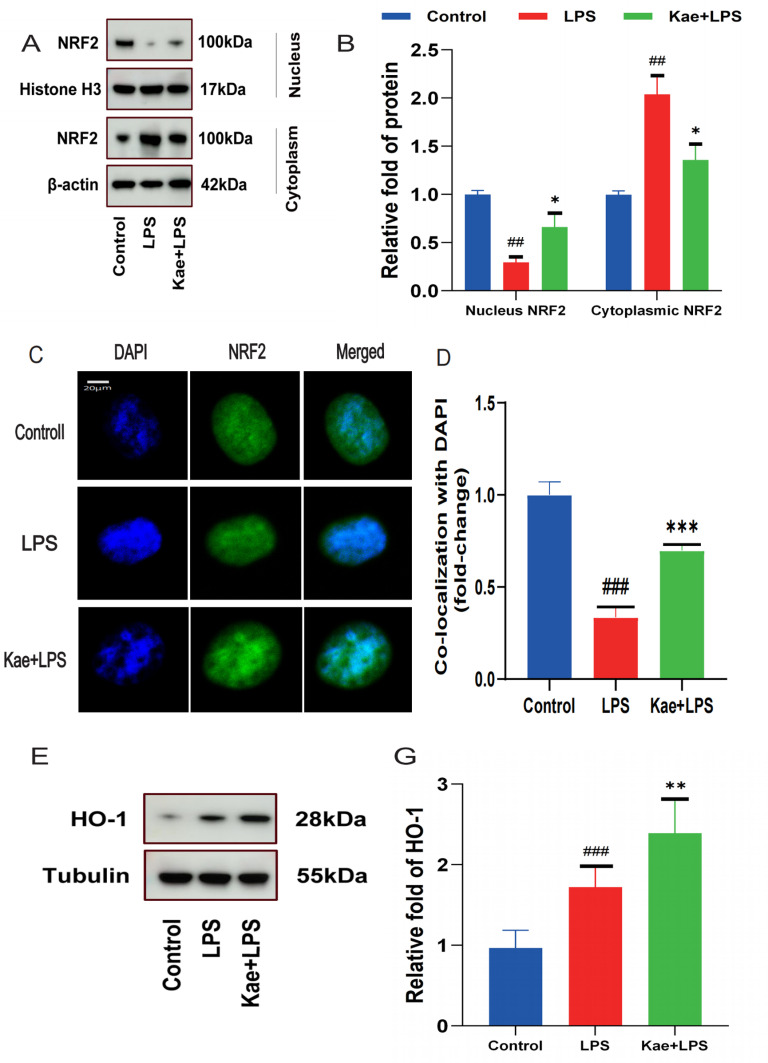
Kaempferol can enhance the activity of NRF2 in macrophages. Cells are divided into three groups. Control group; LPS group; Kae + LPS group, using 1 μg/mL LPS for 4 h; Kae + LPS group, using 100 µM Kae treated macrophages for 2h, followed by 1 μg/ml LPS for 4 h. (A) Extract nuclear and cytoplasmic proteins from macrophages using a protein extraction kit for nuclear and cytoplasmic proteins, and detect the expression levels of NRF2 protein in the nucleus and cytoplasm by Western Blot.(B) Use ImageJ to perform quantitative analysis of NRF2, Histone H3, and β-actin proteins.(C) Observe the levels of NRF2 in the nuclei of different groups of cells using immunofluorescence.(D) Perform quantitative analysis of the immunofluorescence image results using ImageJ.(E) Detect the expression levels of HO-1 and Tubulin proteins in macrophages using Western Blot.(F) Use ImageJ to perform quantitative analysis of HO-1 and Tubulin proteins.“###”The symbol indicates the comparison between the LPS group and the Control group, with a P-value of <0.001.”***”The symbol indicates the comparison between the Kae + LPS group and the LPS group, with a P-value of <0.001.The significance levels are denoted as follows:“#” represents P < 0.05,”##” represents P < 0.01”###” represents P < 0.001,”*” represents P < 0.05,”**” represents P < 0.01,”***” represents P < 0.001,”&” represents P < 0.05,”&&” represents P < 0.01,”&&&” represents P < 0.001.The experimental results were independently repeated three times to ensure the reliability and consistency of the data. Each experiment was conducted under the same conditions and methods to minimize random errors. Kae: Kaempferol; LPS: Lipopolysaccharides; NRF2: Nuclear factor red blood cell 2 related factor 2; HO-1: Heme Oxygenase 1. The error bars shown in the figure represent a 95% confidence interval.

NRF2 plays a key role in regulating intracellular oxidative and antioxidant stress responses, becoming activated upon oxidative stress to maintain cellular oxidative homeostasis. Cytoplasmic and nuclear proteins were extracted from different groups to assess NRF2 expression. Kaempferol decreased NRF2 levels in the cytoplasm but increased NRF2- levels in the nucleus (Fig 4A–B). Total NRF2 levels in macrophages were unchanged in all three groups. In addition, analysis by immunofluorescence also showed that kaempferol increased NRF2 levels in the nucleus (Fig 4C–D).

HO-1 is a downstream protein of NRF2. Upon stimulation, NRF2 was activated, and NRF2 was transferred from the cytoplasm to the nucleus, where it binds to antioxidant response elements (ARE) in the promoter regions of antioxidant genes, regulating the expression of antioxidant enzymes and proteins. This process enhances the antioxidant capacity of the cell, mitigating damage caused by oxidative stress. Because the promoter of the antioxidant enzyme HO-1 is also included in the sequence of the element ARE, NRF2 can enhance the antioxidant capacity by regulating the expression of HO-1. Here, LPS was found to increase HO-1 levels in the LPS group acting alone (it is not contradictory that LPS increased HO-1 levels (Fig 4E–F), which is a cellular self-protection mechanism under inflammatory conditions). Kaempferol plus lipopolysaccharide intervention further increased HO-1 levels. These data suggest that kaempferol activates the NRF2 pathway by promoting NRF2 translocation.

### 3.5. Kaempferol improves inhibition of oxidative stress by activating the NRF2 pathway

**Fig 5:**
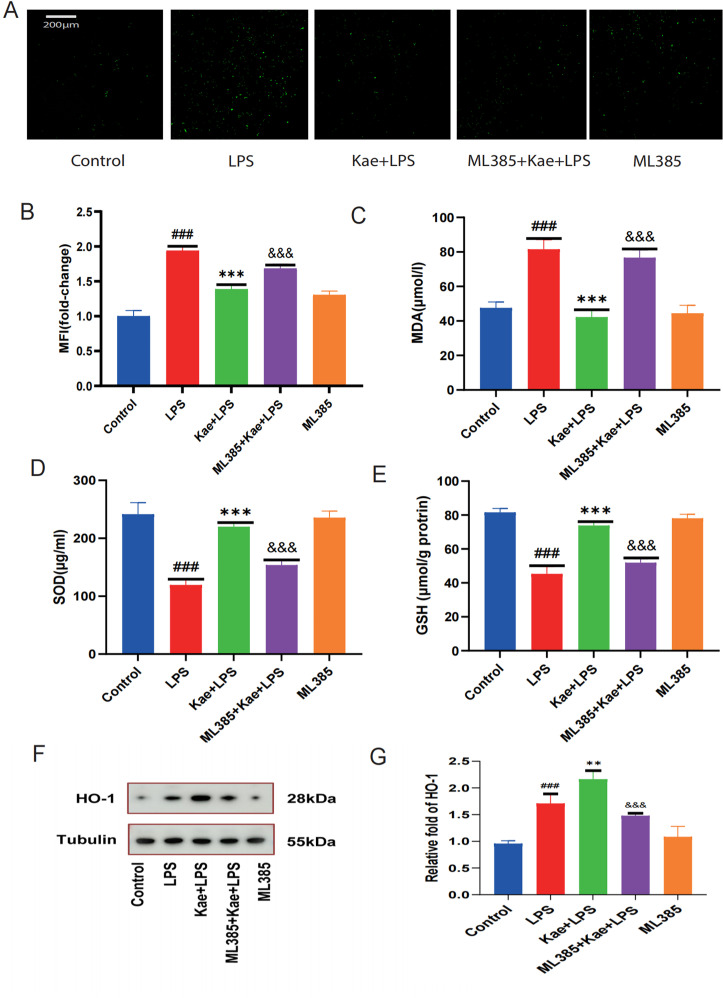
Kaempferol improves oxidative stress by activating the NRF2 pathway. Macrophages are divided into five groups. Control group; LPS group; Kae + LPS group, ML385 + Kae + LPS group, ML385 group. Kae + LPS group,using 100 µM Kae treated macrophages for 2h, followed by 1 μg/ml LPS for 4 h. ML385 + Kae + LPS group; Treat cells with 5 µ MML385 for 1 hour, using 100 µM Kae treated macrophages for 2h, followed by 1 μg/ml LPS for 4 h. (A) Analyze the total ROS levels in cells using the DCFH-DA probe.(B) Perform quantitative analysis of cellular ROS expression using Image J.(C) Measure the expression levels in different groups using the MDA assay kit.(D) Assess the expression levels in different groups using the SOD assay kit.(E) Evaluate the expression levels in different groups using the GSH assay kit.“###”The symbol indicates the comparison between the LPS group and the Control group, with a P-value of <0.001.”***”The symbol indicates the comparison between the Kae + LPS group and the LPS group, with a P-value of <0.001.”&&&”The symbol indicates the comparison between the ML385 + Kae + LPS group and the Kae + LPS group, with a P-value of <0.001.The significance levels are denoted as follows:“#” represents P < 0.05,”##” represents P < 0.01”###” represents P < 0.001,”*” represents P < 0.05,”**” represents P < 0.01,”***” represents P < 0.001,”&” represents P < 0.05,”&&” represents P < 0.01,”&&&” represents P < 0.001.The experimental results were independently repeated three times to ensure the reliability and consistency of the data. Each experiment was conducted under the same conditions and methods to minimize random errors. Kae: Kaempferol; LPS: Lipopolysaccharides; ML385: Specific inhibitor of nuclear factor red blood cell 2 related factor 2 (NRF2). ROS: Reactive oxygen species. SOD: Superoxide dismutase; MDA: Malondialdehyde; GSH: Glutathione; HO-1: Heme Oxygenase 1. The error bars shown in the figure represent a 95% confidence interval.

To confirm whether kaempferol reduces macrophage ROS levels by activating the NRF2 pathway. Macrophages were treated with ML385, a specific inhibitor of NRF2, and compared with the other four groups, it was found that the ML385 + Kae + LPS group significantly increased ROS levels in macrophages (Fig 5A–B), promoted a decrease in MDA levels (Fig 5C), and also led to a decrease in GSH levels, SOD activity, and HO-1 levels (Fig 5D–G). These findings suggest that ML385 diminished the antioxidant effects of kaempferol, highlighting the critical role of the NRF2 pathway in mediating kaempferol’s protective effects.

### 3.6. Kaempferol inhibits macrophage pyroptosis by activating the NRF2 pathway

**Fig 6:**
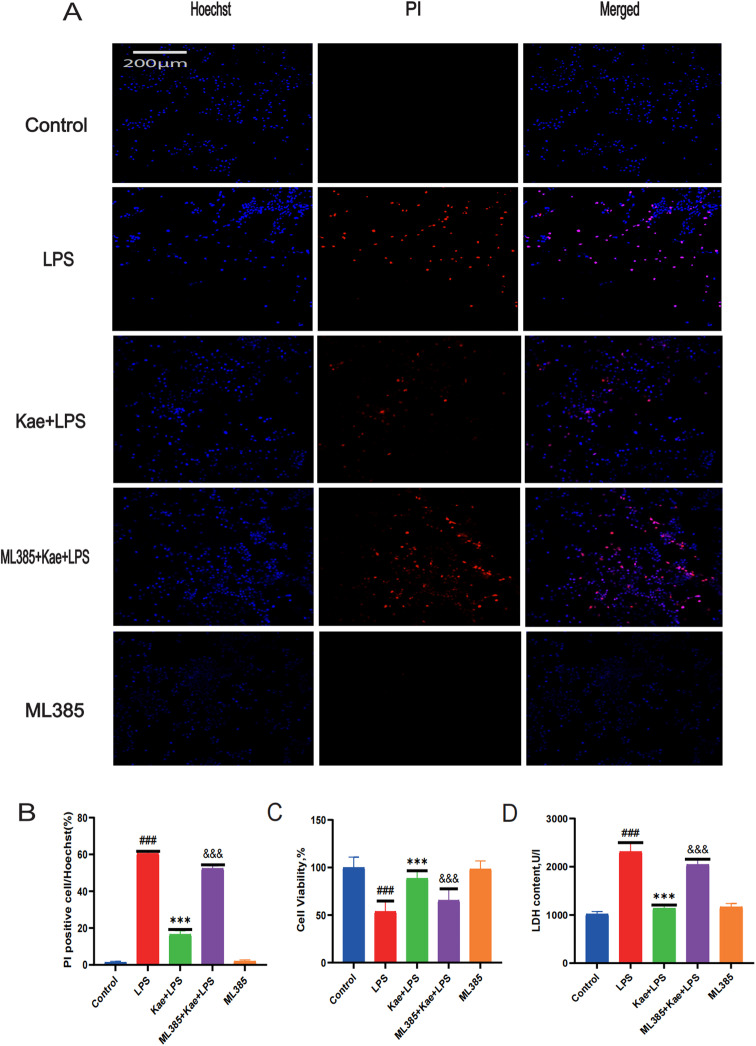
Kaempferol inhibits LPS induced macrophage pyroptosis by activating the NRF2 pathway. Macrophages are divided into 5 groups. Control group; LPS group; Kae + LPS group, ML385 + Kae + LPS group, Kae + LPS group,using 100 µM Kae treated macrophages for 2h, followed by 1 μg/ml LPS for 4 h. ML385 + Kae + LPS group; Treat cells with 5 µ MML385 for 1 hour, using 100 µM Kae treated macrophages for 2h, followed by 1 μg/ml LPS for 4 h. (A) Hochst/PI double staining results, (B) Quantitative analysis using ImageJ, (C) CCK-8 assay results for macrophage viability, (D) Results from the lactate dehydrogenase (LDH) assay kit,“###”The symbol indicates the comparison between the LPS group and the Control group, with a P-value of <0.001.”***”The symbol indicates the comparison between the Kae + LPS group and the LPS group, with a P-value of <0.001.”&&&”The symbol indicates the comparison between the ML385 + Kae + LPS group and the Kae + LPS group, with a P-value of <0.001.The significance levels are denoted as follows:“#” represents P < 0.05,”##” represents P < 0.01”###” represents P < 0.001,”*” represents P < 0.05,”**” represents P < 0.01,”***” represents P < 0.001,”&” represents P < 0.05,”&&” represents P < 0.01,”&&&” represents P < 0.001.The experimental results were independently repeated three times to ensure the reliability and consistency of the data. Each experiment was conducted under the same conditions and methods to minimize random errors. Kae: Kaempferol; LPS: Lipopolysaccharides; Hochst staining; PI: Propyridine iodide; LDH: Lactate dehydrogenase. The error bars shown in the figure represent a 95% confidence interval.

**Fig 7:**
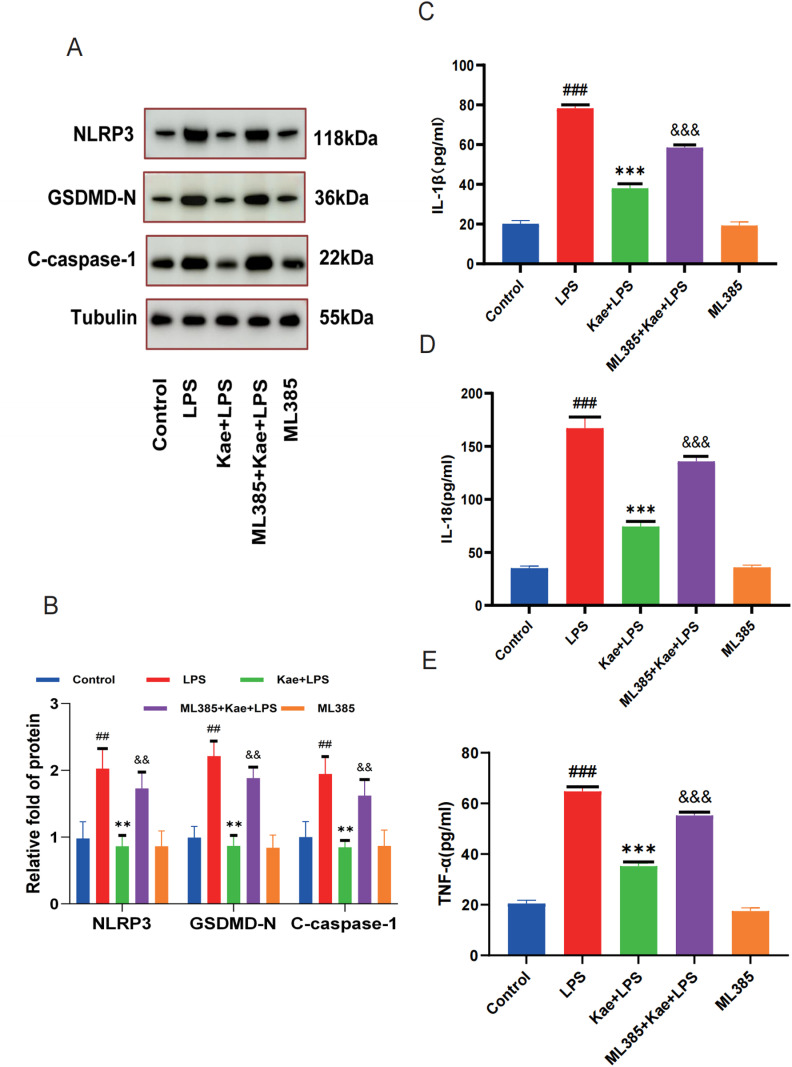
Kaempferol inhibits LPS induced macrophage pyroptosis by activating the NRF2 pathway. Macrophages are divided into 5 groups. Control group; LPS group; Kae + LPS group, ML385 + Kae + LPS group, Kae + LPS group, using 100 µM Kae treated macrophages for 2h, followed by 1 μg/ml LPS for 4 h. ML385 + Kae + LPS group; Treat cells with 5 µ MML385 for 1 hour, using 100 µM Kae treated macrophages for 2h, followed by 1 μg/ml LPS for 4 h. (A) Detection of the expression levels of NLRP3, GSDMD, and Caspase-1 proteins in macrophages by Western Blot. (B) Quantification of NLRP3, GSDMD, and Caspase-1 proteins using ImageJ. (C) Detection of the expression level of the inflammatory cytokine IL-1β in cell supernatants using an ELISA kit. (D) Detection of the expression level of the inflammatory cytokine IL-18 in cell supernatants using an ELISA kit. (E) Detection of the expression level of the inflammatory cytokine TNF-α in cell supernatants using an ELISA kit.“###”The symbol indicates the comparison between the LPS group and the Control group, with a P-value of <0.001.”***”The symbol indicates the comparison between the Kae + LPS group and the LPS group, with a P-value of <0.001.”&&&”The symbol indicates the comparison between the ML385 + Kae + LPS group and the Kae + LPS group, with a P-value of <0.001.The significance levels are denoted as follows:“#” represents P < 0.05,”##” represents P < 0.01”###” represents P < 0.001,”*” represents P < 0.05,”**” represents P < 0.01,”***” represents P < 0.001,”&” represents P < 0.05,”&&” represents P < 0.01,”&&&” represents P < 0.001.The experimental results were independently repeated three times to ensure the reliability and consistency of the data. Each experiment was conducted under the same conditions and methods to minimize random errors. Kae: Kaempferol; LPS: Lipopolysaccharides. The error bars shown in the figure represent a 95% confidence interval.

Reactive oxygen species (ROS) production plays a central role the progression of many inflammatory diseases and is a key mechanism for NLRP3 inflammatory vesicle activation. The above findings have demonstrated that kaempferol can activate NRF2 to reduce the level of oxidative stress in macrophages, however, whether kaempferol can inhibit LPS-induced macrophage pyroptosis through this pathway needs to be further explored. Hoechst/PI staining (Fig 6A–B) and CCK-8 assay (Fig 6C) revealed that ML385 abrogated the protective effect of kaempferol on macrophages. Moreover, the expression of LDH level was significantly increased in the ML385 + Kae + LPS group (Fig 6D). After pretreatment with ML385, it was observed that the expression levels of proteins, such as NLRP3, Caspase1, and GSDMD, were increased in the ML385 + Kae + LPS group (Fig 7A–B), while the ELISA results showed that the levels of inflammatory factors IL-1β, IL-18 and TNF-α were also increased (Fig 7C–E). These data suggest that kaempferol inhibits macrophage pyroptosis by activating the NRF2 pathway.

### 4. Discussion

Atherosclerosis is a common cardiovascular disease that falls under the category of chronic inflammatory diseases. During the progression of the disease, high levels of inflammation are generated in the body, amplifying the inflammatory response cascade [34], which is a key link and major pathophysiological mechanism in cardiovascular atherosclerosis. Research has shown that pyroptosis plays a crucial role in the pathogenesis of atherosclerosis, and pyroptosis is a lytic programmed cell death form triggered by inflammasomes, accompanied by significant inflammatory responses [3]. Therefore, the formation of atherosclerosis is closely related to pyroptosis, making the inhibition of macrophage pyroptosis a potential therapeutic target for preventing cardiovascular diseases. In summary, the discovery of pyroptosis has broadened the understanding of cardiovascular diseases, and targeting macrophage pyroptosis provides a new approach for the treatment of atherosclerosis. This study found that kaempferol effectively inhibits macrophage pyroptosis and alleviates LPS-induced inflammatory responses. Additionally, research has shown that kaempferol reduces oxidative stress by activating the NRF2 pathway, thereby further reducing macrophage pyroptosis.

Macrophages are key mediators of inflammatory responses. They are involved in all stages of atherosclerosis development and progression, from plaque formation to the transition to vulnerable plaques, and are considered important therapeutic targets [35]. Previous studies have shown that increased inflammation not only attracts more circulating monocytes to the atherosclerotic vascular wall but also promotes the development of vulnerable plaque characteristics [36]. After migrating to the arterial vascular wall, monocytes differentiate into pro-inflammatory macrophages, locally amplifying the inflammatory response. Previous research has also shown that if the pro-inflammatory state persists, increased macrophage apoptosis and defective clearance of apoptotic cells occur in the late stages of the disease. This catastrophic combination promotes plaque necrosis, further exacerbating the inflammatory response in atherosclerosis and potentially leading to occlusive luminal thrombosis and its consequences, such as myocardial infarction, stroke, and sudden cardiac death [37]. Therefore, improving macrophage inflammation may be a potential strategy for treating atherosclerosis. However, there is still a lack of molecules that can reduce macrophage inflammation levels during atherosclerosis. In vitro studies have shown that kaempferol may be a key mediator in inhibiting the production of inflammatory factors and alleviating inflammation levels [19]. However, there is still a lack of direct evidence proving kaempferol’s anti-inflammatory effects. This study found that 100µM kaempferol reduced the levels of IL-1β, IL-18, and TNF-α in macrophages, strongly supporting the conclusion of kaempferol’s anti-inflammatory potential.

Pyroptosis is a form of programmed cell death characterized by cell swelling, large bubbles protruding from the plasma membrane, and cell lysis, widely occurring in the initiation, progression, and complications of AS [38]. Pyroptosis requires inflammatory caspase cleavage and activation of the pore-forming effector protein Gasdermin D. The physical rupture of cells leads to the release of pro-inflammatory cytokines IL-1β and IL-18-related molecules, marking the inflammatory potential of pyroptosis [39]. Previous research has focused on endothelial cell pyroptosis in atherosclerotic lesions, with drugs such as melatonin and estrogen inhibiting endothelial cell pyroptosis to reduce AS plaque formation [40]. Additionally, evidence suggests that pyroptosis, as a regulatory necrosis that secretes pro-inflammatory factors, is a significant cause of cell death during the progression of AS [41]. Research has found that kaempferol can improve LPS-induced macrophage viability decline. Macrophage pyroptosis leads to LDH release and PI staining positivity. After kaempferol intervention, LPS-induced LDH release levels and PI staining positivity rates significantly decreased, indicating that kaempferol can alleviate LPS-induced macrophage damage. Since the discovery of the important role of the NLRP3 inflammasome in inflammation-related diseases, the intrinsic mechanisms of NLRP3 inflammasome activation have garnered widespread attention. The NLRP3 inflammasome is a key indicator in the process of pyroptosis [24]. Under inflammatory stimulation, cells activate corresponding inflammasomes based on pathogen-associated molecular patterns (PAMPs) and damage-associated molecular patterns (DAMPs) [42]. Upon activation, the N-terminal PYD domain of NLRP3 serves as a scaffold to promote ASC protein aggregation. ASC contains both PYD and CARD domains. In this process, the PYD of ASC interacts with the PYD of NLRP3, promoting their binding, while the CARD of ASC interacts with pro-caspase-1, recruiting it through the CARD domain. This interaction leads to the self-cleavage of pro-caspase-1, generating mature caspase-1 (p10/p20 tetramer). Activated caspase-1 then recognizes and cleaves the inactive precursors of IL-1β and IL-18, converting them into mature inflammatory cytokines. Additionally, GSDMD produces its N-terminal fragment GSDMD-N. The oligomerization of GSDMD-N leads to the formation of membrane pores, further promoting the release of inflammatory cytokines and cell swelling, ultimately inducing pyroptosis [43]. This study found that kaempferol reduced the expression levels of NLRP3, caspase-1, and GSDMD proteins in macrophages, as well as the expression levels of inflammatory factors such as IL-1β, IL-18, and TNF-α in these cells. Therefore, the results of this study indicate that kaempferol inhibits macrophage pyroptosis by suppressing NLRP3 inflammasome activation.

Inflammation and oxidative stress are closely related and interconnected, being interdependent pathophysiological processes. The mechanisms of pyroptosis and atherosclerosis overlap [44], where oxidative stress not only triggers cell damage but also activates the NLRP3 inflammasome, enhancing the inflammatory response and promoting pyroptosis. Related studies have shown that excessive ROS-induced NLRP3 inflammasome activation leading to pyroptosis is widespread in cardiovascular diseases [45]. Research has found that nicotine promotes ROS production, and this oxidative stress may be an upstream mechanism for NLRP3 inflammasome activation, inducing inflammasome activation and the maturation of inflammatory cytokines, subsequently leading to pyroptosis and cell death [46]. Research has found that quercetin can inhibit macrophage pyroptosis by inhibiting oxidative stress production [47]. This study found that LPS-treated macrophages had increased intracellular ROS levels, and kaempferol alleviated this phenomenon. The study also found that kaempferol reduced MDA levels in LPS-treated macrophages. In addition, chrysin enhanced the activity of the macrophage antioxidant system, as evidenced by increased levels of GSH, HO-1, and SOD. However, LPS treatment alone reduced the expression of the nuclear factor NRF2 while increasing HO-1 levels. This difference can be explained by two factors: first, HO-1 is regulated not only by NRF2 but also by multiple transcription factors [48]. Second, LPS treatment induces redox imbalance, leading to negative feedback regulation within the cells, resulting in increased HO-1 expression.

Next, we will evaluate the potential mechanisms by which kaempferol enhances the oxidative stress response. Reports indicate that NRF2, a classic transcription factor, regulates the transcription of various antioxidant proteins, including HO-1. The transcriptional response of NRF2 is crucial for maintaining body balance [49]. After LPS treatment, the expression level of NRF2 in the cytoplasm increased, while it decreased in the nucleus. However, kaempferol reversed this effect, leading to a significant increase in HO-1 levels. These results suggest that kaempferol promotes NRF2 nuclear translocation, thereby improving the oxidative stress response. Studies have shown that perfluorooctanol alleviates the imbalance of inflammatory responses by inhibiting NLRP3 inflammasome activation and reduces oxidative stress through the Nrf2/HO-1 signaling pathway in LPS-induced macrophages [50]. Kaempferol activates the nuclear factor-E2-related factor 2 (Nrf2)/SLC7A11/GPX4 signaling pathway, enhancing antioxidant capacity, but after NRF2 inhibition, kaempferol’s antioxidant capacity against oxygen-glucose deprivation/reperfusion (OGD/R)-induced neuronal damage decreases [51]. This study found that using the ML385 inhibitor alone did not affect the levels of NLRP3, Caspase-1, and GSDMD. Research has also reported that treating macrophages with 5µM ML385 alone does not cause inflammatory changes [32]. To further validate this hypothesis, macrophages were pre-treated with the NRF2 nuclear translocation inhibitor ML385, which significantly reduced the antioxidant stress response induced by kaempferol. Additionally, the increased in macrophage activity and reduced NLRP3 inflammasome activity triggered by kaempferol were also offset by ML385. These findings strongly support the conclusion that kaempferol alleviates oxidative stress and macrophage pyroptosis by activating the NRF2 pathway.

In summary, kaempferol exhibits anti-inflammatory and antioxidant effects, playing a key role in inflammation prevention. It effectively inhibits oxidative stress through the NRF2 pathway, thereby alleviating macrophage pyroptosis. The study also found that kaempferol’s protective effects on macrophage pyroptosis through the NRF2 pathway provide new insights for future research on the application of kaempferol in the treatment of atherosclerosis. Although this study evaluated the protective effects of kaempferol on LPS-induced macrophage pyroptosis in vitro, these data do not directly confirm whether kaempferol can stabilize atherosclerosis by inhibiting macrophage pyroptosis, which is a limitation of this study. Therefore, further animal experiments and mechanistic studies are needed to verify its efficacy and safety, i.e., to explore the effects of kaempferol on macrophage pyroptosis and its potential mechanisms in animal models. Future research should focus on the anti-inflammatory and antioxidant effects of kaempferol in animal models and its impact on plaque stability, providing a more solid experimental basis for the clinical treatment of atherosclerosis.

## Supporting information

S1 Data Raw data for all figures. (XLSX)
